# Clinical Significance of PIK3CA Gene in Non-Small-Cell Lung Cancer: A Systematic Review and Meta-Analysis

**DOI:** 10.1155/2020/3608241

**Published:** 2020-08-17

**Authors:** Yi Wang, Yan Wang, Jialong Li, Jue Li, Guowei Che

**Affiliations:** ^1^Department of Thoracic Surgery, The First People's Hospital of Neijiang, Neijiang Affiliated Hospital of Chongqing Medical University, Neijiang, 641000 Sichuan, China; ^2^Department of Thoracic Surgery, West China Hospital, Sichuan University, Chengdu 610041, China

## Abstract

**Aim:**

To explore the clinicopathological and prognostic role of PIK3CA gene mutation and expression in non-small-cell lung cancer (NSCLC) patients.

**Methods:**

A systematic and comprehensive literature search was conducted through EMBASE (via OVID), Web of Science, and PubMed. Relative risks (RRs) and hazard ratios (HRs) with 95% confidence intervals (CIs) were combined to evaluate the relationship of the PIK3CA gene with clinicopathological parameters and the survival of NSCLC patients, respectively.

**Results:**

A total of 13 studies involving 3908 patients were analyzed in our study. Only lymph node metastasis status had an association with PIK3CA mutation (RR = 2.823; 95% CI: 1.128-7.065; *P* = 0.029). The results indicated that PICK3CA mutation was related with overall survival (OS) (HR = 1.55; 95% CI: 1.13-2.13; *P* = 0.007), progression-free survival (PFS) (HR = 1.48; 95% CI: 1.06-2.08; *P* = 0.023), and cancer-specific survival (CSS) (HR = 2.63; 95% CI: 1.00-6.92; *P* = 0.005). Furthermore, PIK3CA high expression was more prevalent in NSCLC patients with smoking history (RR = 2.42; 95% CI: 1.04-5.61; *P* = 0.040). However, no significant relation between PIK3CA expression and OS was found (HR = 0.80; 95% CI: 0.58-1.12; *P* = 0.193).

**Conclusion:**

PIK3CA mutation may affect lymph node metastasis and serve as a promising prognostic factor, and smoking may be related with PIK3CA high expression in NSCLC patients. However, more well-designed prospective researches are needed to verify the abovementioned findings.

## 1. Introduction

The lipid kinase phosphatidylinositol-3 kinase (PI3K) family is involved in a number of normal cellular processes. For example, it plays an important role in the activation of serine/threonine kinase AKT, which activates a lot of factors like mTOR [1]. The PI3K-AKT-mTOR pathway is crucial in the domination of multiple tumor-relevant regulatory processes such as cell growth, survival, and cycle progression; meanwhile, somatic mutations in this pathway are more prevalent in tumors [2, 3]. PI3K consists of a regulatory (p85) subunit and a catalytic (p110) subunit; the latter one is encoded by 3 genes including PIK3CA, PIK3CB, and PIK3CD, and the PIK3CA mutation is the most frequent in cancers [4].

PIK3CA mutation has been found in a large variety of human tumors, and a frequency of 2-7% in non-small-cell lung cancer (NSCLC) was observed according to previous studies [5, 6]. However, the clinical role of PIK3CA mutation in NSCLC patients is still debated. Whether PIK3CA gene mutation has a significant relation with clinicopathological characteristics such as age, differentiation, and lymph node metastasis and whether it could affect the survival of NSCLC patients are unclear up to now.

Furthermore, the PIK3CA gene has been recognized as a candidate driver gene of lung squamous cell carcinoma and may contribute to the tumor cell growth and development of NSCLC [7–10]. However, the clinicopathological and prognostic roles of the PIK3CA gene overexpression have not been identified accurately due to limited publications about this field. Whether PIK3CA gene expression status could help with the evaluation of disease progression and prediction of survival in NSCLC needs more investigation.

Thus, we conducted the current systematic review and meta-analysis to further clarify the clinical value of PIK3CA mutation and expression status in NSCLC, which may contribute to the formulation of therapy strategy and management of NSCLC patients in the future.

## 2. Materials and Methods

This meta-analysis was conducted according to the Preferred Reporting Items of Systematic Reviews and Meta-Analysis (PRISMA) guidelines [11].

### 2.1. Literature Search

A systematic and comprehensive literature retrieval was conducted through the EMBASE (via OVID), Web of Science, and PubMed databases from the date of database establishment to September 25, 2019. The following key words were used: “PIK3CA,” “lung,” “pulmonary,” “cancer,” “tumor,” “carcinoma,” and “neoplasm.” MeSH terms and free texts were applied to increase sensitivity. Furthermore, the references cited in the included studies were also reviewed for eligibility.

### 2.2. Inclusion and Exclusion Criteria

The following inclusion criteria were applied in this meta-analysis: (1) patients were diagnosed with NSCLC pathologically; (2) patients were divided into different groups and compared according to the status of PIK3CA mutation or expression; (3) the association between the PIK3CA gene and the survival of NSCLC patients was assessed and represented as the hazard ratios (HRs) with 95% confidence intervals (CIs) or Kaplan-Meier's survival curves; (4) for overlapped or duplicated data, only the latest one was included; and (5) studies must have a Newcastle − Ottawa quality assessment scale (NOS) score ≥ 6.

The following exclusion criteria were applied in the current meta-analysis: (1) reviews, letters, case reports, animal trials, or conference abstracts and (2) insufficient data for the calculation of HRs with 95% CIs.

### 2.3. Data Extraction and Research Quality Assessment

The following information was extracted from the included studies: the name of the first author, publication year, country, sex, age, smoking history, sample size, the number of patients with PIK3CA mutation or expression, pathological type, lymph node metastasis status, differentiation status, tumor-node-metastasis (TNM) stage, therapy strategy, clinical outcome including overall survival (OS), progression-free survival (PFS) and cancer-specific survival (CSS), source of HR, NOS score, relative risk (RR) with 95% CI, and HR with 95% CI.

The quality of the included studies was evaluated as we previously described [12].

The literature retrieval, selection, data extraction, and study quality assessment were all performed by two authors independently (Yi Wang and Yan Wang).

### 2.4. Statistical Analysis

The statistical methods used in this article are similar to those we described before [12]. The STATA version 12.0 software was applied for statistical analysis in this meta-analysis. The association of PIK3CA mutation and expression with clinical pathological parameters was evaluated by the combined RRs with 95% CIs, and the relationship of PIK3CA with survival in NSCLC patients was assessed by the combined HRs with 95% CIs. The HRs with 95% CIs from the multivariable models were applied whenever available; if the publications did not report them directly, they would be estimated from the Kaplan-Meier survival curves through the method introduced by Tierney et al. [13]. The Chi-square-based *Q*-test and *I*^2^ statistic were performed to calculate the heterogeneity among the included studies [14]. If significant heterogeneity was observed, represented as *P* < 0.10 or/and *I*^2^ > 50%, the random-effect model was applied; otherwise, the fixed-effect model was used [15]. The stability of the pooled results was evaluated by the sensitivity analysis. The potential publication bias was calculated using Begg's funnel plot and Egger's test. A log-rank *P* value < 0.05 was regarded as statistically significant in our meta-analysis.

## 3. Results

### 3.1. Literature Retrieval

**Figure 1:**
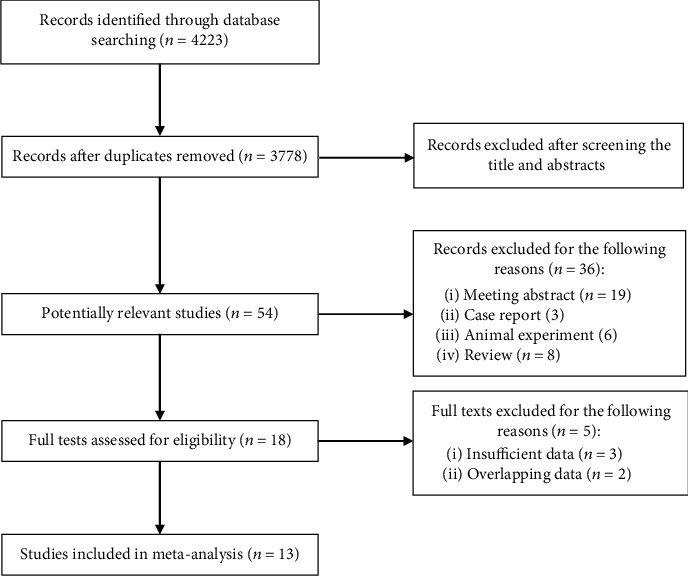
The flow diagram of this meta-analysis.

After searching three electronic databases, 4223 records were identified. Then, 3778 records were reviewed for relativity after removing 445 duplicates, and 54 publications were found to be potentially relevant. Eighteen full text publications were assessed for eligibility, and a total of 13 studies were included in this meta-analysis after excluding 5 studies because of overlapping or insufficient data [16–28] (Figure 1).

### 3.2. Basic Characteristics of Included Studies

Thirteen researches involving 3908 patients were analyzed. About half of them (6/13) were from China. The sample size ranged from 52 to 1117. The ratio for patients with PIK3CA mutation and high expression ranged from 2.8% to 19.7% and from 29.2% to 78.9%, respectively. Ten and three studies reported PIK3CA mutation and expression separately. Other specific information is presented in Table 1.

### 3.3. Association of PIK3CA Mutation with Clinicopathological Characteristics in NSCLC

Based on the information reported by 10 included studies [17, 18, 21–28], we explored the association of PIK3CA gene mutation with the sex (male vs. female), age (≥60 vs. <60), lymph node metastasis (+ vs. -), TNM stage (advanced vs. limited), and smoking history (yes vs. no). Unfortunately, only lymph node metastasis status seemed to have a relation with PIK3CA mutation (RR = 2.823, 95% CI: 1.128-7.065, *P* = 0.029) (Table 2).

### 3.4. Association of PIK3CA Expression with Clinicopathological Characteristics in NSCLC

According to the data provided by 3 included studies [16, 19, 20], we explored the association of PIK3CA gene expression with sex (male vs. female), age (≥65 vs. <65), differentiation (lower vs. higher), lymph node metastasis (+ vs. -), TNM stage (advanced vs. limited), and smoking history (yes vs. no). Only smoking was found to be significantly related with PIK3CA high expression (HR = 2.42; 95% CI: 1.04-5.61; *P* = 0.040; *I*^2^ = 23.95%; *P*_heterogeneity_ = 0.252) (Table 3).

### 3.5. Association of PIK3CA with Survival of NSCLC Patients

**Figure 2:**
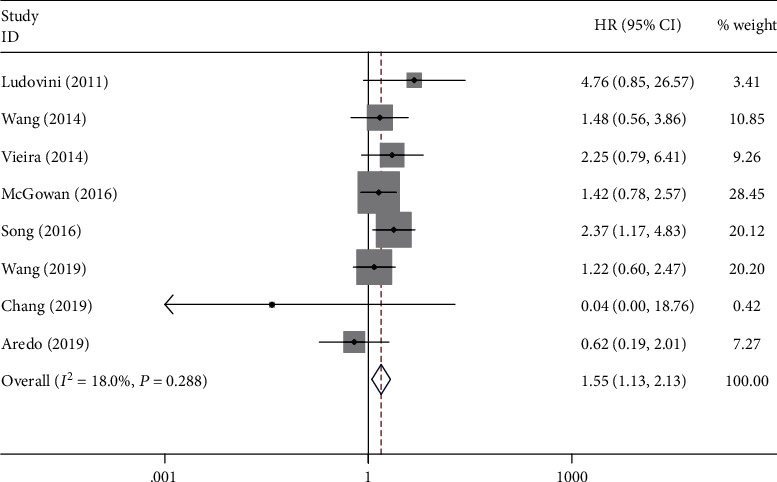
Forest plot of the association between PIK3CA mutation and overall survival.

**Figure 3:**
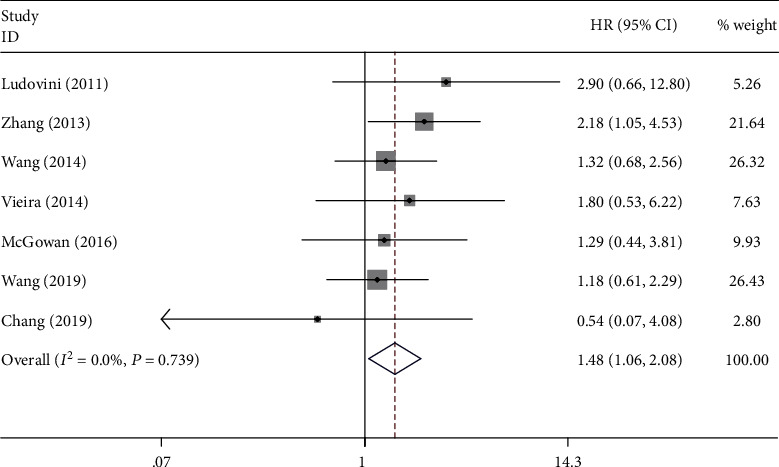
Forest plot of the association between PIK3CA mutation and progression-free survival.

A total of 10 studies involving 3364 patients reported the prognostic value of PIK3CA mutation in NSCLC. The pooled results indicated that PIK3CA mutation was significantly associated with poor OS (HR = 1.55; 95% CI: 1.13-2.13; *P* = 0.007; *I*^2^ = 18%; *P*_heterogeneity_ = 0.288) (Figure 2) and PFS (HR = 1.48; 95% CI: 1.06-2.08; *P* = 0.023; *I*^2^ = 0.0%; *P*_heterogeneity_ = 0.739) (Figure 3). Furthermore, Imperatori et al. reported that NSCLC patients with PIK3CA mutation had poorer CSS (HR = 2.63; 95% CI: 1.00-6.92; *P* = 0.05).

**Figure 4:**
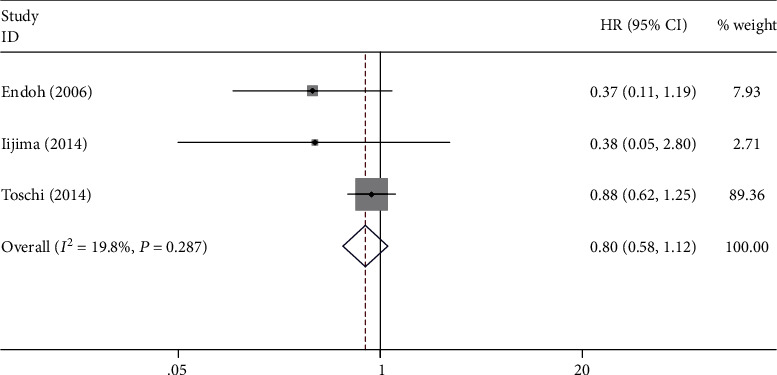
Forest plot of the association between PIK3CA expression and overall survival.

A total of 3 studies involving 544 patients explored the prognostic role of PIK3CA expression in NSCLC. No significant relation between OS and PIK3CA high expression was observed (HR = 0.80; 95% CI: 0.58-1.12; *P* = 0.193; *I*^2^ = 19.8%, *P*_heterogeneity_ = 0.287) (Figure 4, Table 4).

### 3.6. Sensitivity Analysis and Publication Bias

**Figure 5:**
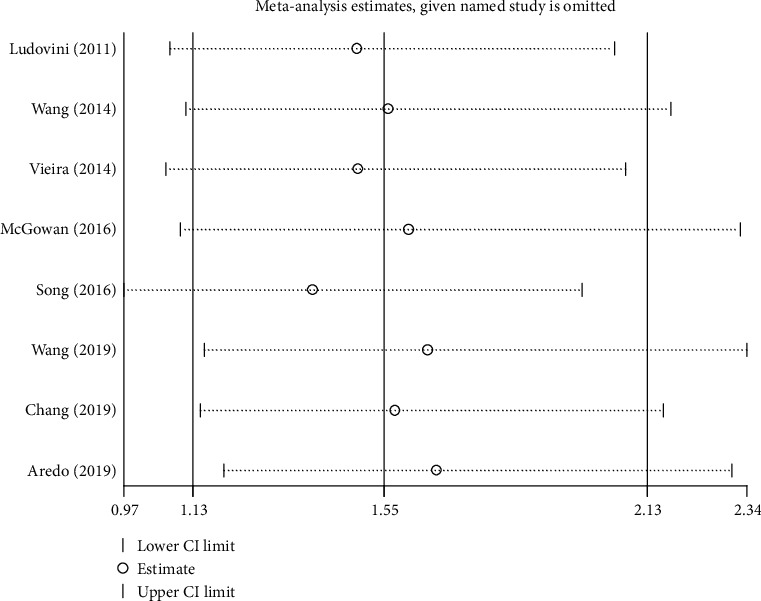
Sensitivity analysis of the association between PIK3CA mutation and overall survival.

**Figure 6:**
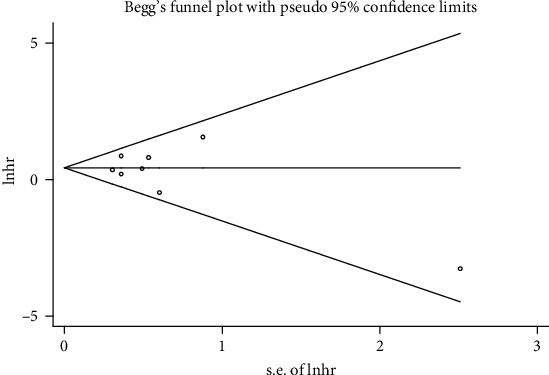
Begg's funnel plot of the association between PIK3CA mutation and overall survival.

The sensitivity analysis indicated that the study of Song et al. [24] had a significant influence on the pooled results (Figure 5). Begg's funnel plot was symmetrical, and the *P* value of Egger's test was 0.54, which both indicated that no significant publication bias existed (Figure 6).

## 4. Discussion

Based on previous studies, PIK3CA mutation occurred more in lung squamous cell carcinoma with a frequency of 11.4% [23] than in lung adenocarcinoma with a frequency of 2.8% [24]. The most common mutation hotspots of the PIK3CA gene are located in the helical domain, especially E545K and E542K on exon 9, and the less frequent mutation hotspots of PIK3CA are located in the kinase domain like H1047R on exon 20 [29]. The mutations in helical domains are thought to cause an activating effect through “unlocking” inactivated conformations, but the mutations in kinase domains change the interaction between the cellular membrane and protein and then provide access to the kinase substrate [30].

Although PIK3CA mutations are relatively common in cancers, especially in NSCLC [21–28], whether they play a role in the tumor development and prognosis of NSCLC patients indeed remains controversial up to now. Among the 10 included studies which focused on PIK3CA mutation in NSCLC, more than half of them did not provide detailed information for us to calculate the relation of PIK3CA gene mutation with sex, age, lymph node metastasis, TNM stage, and smoking [21, 22, 25–28]. Based on remaining studies, we manifested that PIK3CA gene mutation did not show a significant relation with the sex, age, TNM stage, or smoking. Interestingly, Zhang et al. reported a positive relationship between PIK3CA mutation and lymph node metastasis (RR = 2.823; 95% CI: 1.128-7.065; *P* = 0.029). It has been demonstrated that the activation of the PI3-kinase pathway might enhance the invasive ability of tumor cells into lymph nodes and PIK3CA mutation is related with the loss of PTEN which inhibits cell migration [31]. However, more detailed mechanisms remain unclear up to now.

The pooled results in our study indicated that PIK3CA mutation was an independent risk factor for OS and PFS for NSCLC patients. Actually, only Song et al. [24] and Zhang et al. [18] reported the positive relation of PIK3CA mutation with OS (HR = 2.37; 95 CI: 1.17-4.83) and PFS (HR = 2.18; 95% CI: 1.05-4.53) separately, which meant most of the relevant studies manifested that PIK3CA mutation did not affect the survival of NSCLC patients significantly. Interestingly, both of the studies performed by Song et al. and Zhang et al. only enrolled lung adenocarcinoma patients who underwent surgical resection. Therefore, our findings about the prognostic value of PIK3CA gene mutation may be confined to this population. About the mechanisms by which PIK3CA gene mutation affects the prognosis of NSCLC patients, there are several potential causes. First, the mutation of PIK3CA signaling pathways might result in the resistance to epidermal growth factor receptor-tyrosine kinase inhibitors (EGFR-TKIs) in NSCLC patients [5]. Second, PIK3CA mutation activates AKT through phosphorylation and the pAKT expression has been demonstrated to be related with poor prognosis [32]. Third, the PI3-kinase pathway activation may enhance the invasion of lymph nodes by tumor cells and PIK3CA mutation is related with the loss of PTEN which inhibits cell migration [31].

Few studies focused on the clinical role of PIK3CA expression status in NSCLC in the past years. Toschi et al. reported that PIK3CA high expression was more prevalent in patients who were male or with lower differentiation degree [20]; however, Iijima et al. demonstrated the insignificant association between PIK3CA expression and sex and differentiation [19], which is consistent with our conclusions. Meanwhile, Toschi et al. manifested that patients with smoking history were more likely to experience PIK3CA high expression than nonsmoking patients [20], and we proved this in the current study, which is inconsistent with the results reported by Iijima et al. [19]. Actually, the patients they analyzed were from different countries and with different pathological types and tumor stages and the detailed mechanisms were unclear. Thus, more studies from other countries or regions with bigger sample sizes are required to further clarify the abovementioned findings.

Our study showed that PIK3CA expression status was not an independent risk factor for OS of NSCLC patients (HR = 0.80; 95% CI: 0.58-1.12; *P* = 0.193), which is consistent with the results of 3 included studies [16, 19, 20]. This indicated that the expression status of the PIK3CA gene may not show an obvious connection with the disease progress of NSCLC patients.

Overall, the clinical value of the PIK3CA gene in NSCLC remains controversial and a number of fields need more valuable investigation. In addition to the aspects mentioned above, we suggest that it would make sense to explore the prognostic role of the PIK3CA gene in advanced stage NSCLC patients who receive chemoradiotherapies as primary treatment. In detail, we could investigate whether the mutation or expression status of the PIK3CA gene plays a role in formulating the treatment protocol and predicting the therapeutic effect. Furthermore, a comparison of PIK3CA mutation between lung adenocarcinoma and lung squamous cell carcinoma would be necessary because we have found that PIK3CA mutation occurred more frequently in smoking patients and it is widely known that the occurrence of squamous cell carcinoma is closely related to smoking. The detailed interactions of smoking with PIK3CA expression are also unclear. Besides, Wang et al. manifested that PIK3CA mutation frequently coexisted with EGFR/KRAS mutations and among 34 PIK3CA mutant patients; 17 and 4 cases coexisted with EGFR mutation and KRAS mutation, respectively [21]. Unfortunately, other included studies did not report the correlation of PIK3CA with EGFR, KRAS, or BRAF mutations, although some of them explored the clinical role of these genes in NSCLC. So, the interaction of PIK3CA with these genes is worth exploring.

There are some limitations in this study. First, all included studies are retrospective and only 2 studies enrolled more than 500 patients. Second, due to lack of original data, we could not perform subgroup analyses stratified by pathological type, age, TNM stage, and so on. Third, we did not verify the results reported in this study using the data in our hospital because the detection of PIK3CA gene mutation or expression status is not a regular test for most NSCLC patients admitted to our hospital.

## 5. Conclusion

In conclusion, PIK3CA mutation might affect lymph node metastasis and serve as a promising prognostic factor, and smoking may be related with PIK3CA high expression in NSCLC patients. However, more well-designed prospective studies are still needed to verify our findings.

## Figures and Tables

**Table 1:** Basic characteristics of included studies.

Author	Year	Country	Sample size	Positive, *n* (%)	Histology type	Treatment	TNM stage	PIK3CA status	Outcome	Source of HR	NOS score
Endoh [16]	2006	Japan	52	26 (50)	NSCLC	Surg	I-IV	High expression	OS	R	6
Ludovini [17]	2011	Italy	145	6 (4.1)	NSCLC	Non-surg	III-IV	Mutation	OS/PFS	R	7
Zhang [18]	2013	China	122	24 (19.7)	AC	Surg	I-IV	Mutation	PFS	E	8
Iijima [19]	2014	China	57	45 (78.9)	SCC	Surg	I-III	High expression	OS	E	7
Toschi [20]	2014	Italy	435	127 (29.2)	NSCLC	Surg	I-IV	High expression	OS	E	7
Wang [21]	2014	China	1117	34 (3.0)	NSCLC	Surg	I-IV	Mutation	OS/PFS	E	7
Vieira [22]	2014	France	60	5 (8.3)	SMC	Surg	I-IV	Mutation	OS/PFS	R	7
McGowan [23]	2016	Norway	308	35 (11.4)	SCC	Surg	I-III	Mutation	OS	E	7
Song [24]	2016	China	810	23 (2.8)	AC	Surg	I-IIIA	Mutation	OS	R	8
Imperatori [25]	2017	Italy	167	5 (3.0)	NSCLC	Surg	I	Mutation	CSS	R	6
Wang [26]	2019	China	416	NR	NSCLC	Non-surg	III-IV	Mutation	OS/PFS	R	7
Chang [27]	2019	China	33	1 (3.0)	NSCLC	Non-surg	IV	Mutation	OS/PFS	R	7
Aredo [28]	2019	America	186	7 (3.8)	NSCLC	Mixed	NR	Mutation	OS	R	6

NSCLC: non-small-cell lung cancer; AC: adenocarcinoma; SCC: squamous cell carcinoma; SMC: sarcomatoid carcinoma; Surg: surgery; Non-surg: nonsurgery; TNM: tumor-node-metastasis; OS: overall survival; PFS: progression-free survival; CSS: cancer-specific survival; HR: hazard ratio; NR: not reported; R: reported; E: estimated; NOS: Newcastle-Ottawa quality assessment scale.

**Table 2:** Associations of PIK3CA mutation with clinicopathological characteristics in non-small-cell lung cancer.

Author	Sex (M vs. F)	Age (≥60 vs. <60)	Lymph node metastasis (*N*+ vs. -)	TNM (advanced vs. limited)	Smoking history (yes vs. no)
Ludovini [17]	0.406 (0.077-2.148)				0.709 (0.148-3.384)
Zhang [18]		0.691 (0.333-1.434)	2.823 (1.128-7.065)	2.083 (0.890-4.877)	1.109 (0.532-2.314)
Wang [21]	—	—	—	—	—
Vieira [22]	—	—	—	—	—
McGowan [23]	1.080 (0.551-2.118)			0.974 (0.399-2.378)	—
Song [24]	1.341 (0.587-3.063)	1.473 (0.654-3.321)		0.962 (0.427-2.167)	
Imperatori [25]	—	—	—	—	—
Wang [26]	—	—	—	—	—
Chang [27]	—	—	—	—	—
Aredo [28]	—	—	—	—	—
Overall (RR (95% CI), *P*; *I*^2^, *P*_heterogeneity_)	1.07 (0.65-1.76), 0.789; 0.0, 0.452	0.97 (0.56-1.67), 0.910; 45.8, 0.174	—	1.25 (0.76-2.04), 0.375; 4.0, 0.353	1.02 (0.53-1.99), 0.947; 0.0, 0.612

RR: relative risk; CI: confidence interval; M: male; F: female; TNM: tumor-node-metastasis.

**Table 3:** Associations of PIK3CA expression with clinicopathological characteristics in non-small-cell lung cancer.

Author	Sex (M vs. F)	Age (≥65 vs. <65)	Differentiation (lower vs. higher)	Lymph node metastasis (*N*+ vs. -)	TNM (advanced vs. limited)	Smoking history (yes vs. no)
Endoh [16]	—	—		—	—	—
Iijima [19]	1.241 (0.180-8.546)	0.981 (0.236-4.083)	0.627 (0.195-2.021)	0.703 (0.200-2.472)	0.627 (0.195-2.021)	0.900 (0.136-5.945)
Toschi [20]	2.232 (1.267-3.934)		1.384 (1.035-1.851)		0.886 (0.654-1.200)	3.091 (1.208-7.911)
Overall (RR (95% CI), *P*; *I*^2^, *P*_heterogeneity_)	1.39 (0.24-7.89), 0.714; 0.0, 0.796	—	1.32 (1.00-1.75), 0.053; 39.7, 0.198	—	0.87 (0.65-1.16), 0.34; 0.0, 0.575	2.42 (1.04-5.61), 0.040; 23.9, 0.252

RR: relative risk; CI: confidence interval; M: male; F: female; TNM: tumor-node-metastasis.

**Table 4:** Meta-analyses for the association of PIK3CA with survival of non-small-cell lung cancer patients.

Analysis	No. of studies	HR (95% CI)	Log-rank *P* value	*I* ^2^ (%)	*P* value
Mutation					
Overall survival	8	1.55 (1.13-2.13)	0.007	18.0	0.288
Progression-free survival	7	1.48 (1.06-2.08)	0.023	0.0	0.739
Cancer-specific survival	1	2.63 (1.00-6.92)	0.05	—	—
Expression					
Overall survival	3	0.80 (0.58-1.12)	0.193	19.8	0.287

HR: hazard ratio; CI: confidence interval.
